# Exogenous hepatitis B virus envelope proteins induce endoplasmic reticulum stress: involvement of cannabinoid axis in liver cancer cells

**DOI:** 10.18632/oncotarget.7950

**Published:** 2016-03-07

**Authors:** Roberta Montalbano, Birgit Honrath, Thaddeus Till Wissniowski, Moritz Elxnat, Silvia Roth, Matthias Ocker, Karl Quint, Yuri Churin, Martin Roederfeld, Dirk Schroeder, Dieter Glebe, Elke Roeb, Pietro Di Fazio

**Affiliations:** ^1^ Department of Visceral, Thoracic and Vascular Surgery, Philipps University of Marburg, Marburg, Germany; ^2^ Division of Gastroenterology, Philipps University of Marburg, Marburg, Germany; ^3^ Institute for Surgical Research, Philipps University of Marburg, Marburg, Germany; ^4^ Department of Gastroenterology, Justus Liebig University, Giessen, Germany; ^5^ Institute of Medical Virology, National Reference Centre for Hepatitis B and D Viruses, Justus Liebig University, Giessen, Germany; ^6^ Present address: Department of Gastroenterology CBF, Charité University Medicine Berlin and Bayer Pharma AG, Experimental Medicine Oncology, Berlin, Germany

**Keywords:** hepatitis B virus, endoplasmic reticulum stress, endocannabinoid system

## Abstract

HBV represents the most common chronic viral infection and major cause of hepatocellular carcinoma (HCC), although its exact role in liver tumorigenesis is unclear. Massive storage of the small (SHBs), middle (MHBs) and large surface (LHBs) HBV envelope proteins leads to cell stress and sustained inflammatory responses. Cannabinoid (CB) system is involved in the pathogenesis of liver diseases, stimulating acute and chronic inflammation, liver damage and fibrogenesis; it triggers endoplasmic reticulum (ER) stress response. The aim of our work was to investigate the activation of ER stress pathway after ectopic HBV envelope proteins expression, in liver cancer cells, and the role exerted by CB receptors. PCR, immunofluorescence and western blotting showed that exogenous LHBs and MHBs induce a clear ER stress response in Huh-7 cells expressing CB1 receptor. Up-regulation of the chaperone BiP/GRP78 (Binding Immunoglobulin Protein/Glucose-Regulated Protein 78) and of the transcription factor CHOP/GADD153 (C/EBP Homologous Protein/Growth Arrest and DNA Damage inducible gene 153), phosphorylation of PERK (PKR-like ER Kinase) and eIF2α (Eukaryotic Initiation Factor 2α) and splicing of XBP1 (X-box binding protein 1) was observed. CB1^−/−^ HepG2 cells did not show any ER stress activation. Inhibition of CB1 receptor counteracted BiP expression in transfected Huh-7 and in HBV^+^ PLC/PRF/5 cells; whereas no effect was observed in HBV^−^ HLF cells. These results suggest that HBV envelope proteins are able to induce the ER stress pathway. CB1 expression is directly correlated with ER stress function. Further investigations are needed to clarify the involvement of cannabinoid in HCC progression after HBV infection.

## INTRODUCTION

Since 30 years chronic infection with HBV leading to liver cirrhosis has been linked epidemiologically to the development of HCC. Although several mechanisms of a direct and indirect hepatocarcinogenic role of HBV have been hypothesized, the precise ways by which HBV infection triggers HCC is not completely known and a wide understanding of HBV infection effects is needed.

HBV is a member of the *Hepadnaviridae* family, with a small circular partially double stranded DNA genome, containing four overlapping open reading frames encoding for the core protein, forming the viral capsid, for the small (SHBs), medium (MHBs) and large (LHBs) envelope proteins, forming subviral particles, for the viral polymerase and for the protein HBx [1]. Several studies demonstrated a strong connection between the transcriptional activator HBx, able to trans-activate the expression of numerous cellular and viral genes, and liver cancer [2–4]. Moreover, the excessive production and accumulation of the HBV envelope proteins, or truncated forms of the MHBs and LHBs proteins, have been ascribed to have oncogenic potential, increasing cell proliferation and strong ER stress [5]. It is known since long time that liver of people with chronic HBV infection frequently contains individual hepatocytes with accumulated LHBs, causing the formation of so called ground glass hepatocytes, with altered ER structures [1, 6]. Moreover, liver damage and subsequent HCC occurred in mice overexpressing the LHBs protein in hepatocytes [5]. Despite many different groups have described so far a link between HBV envelope proteins and stress cellular pathways, like ER stress, and have speculated about a connection with HCC evolution, the molecular pathways underlying these processes have not been fully elucidated yet.

The unfolded protein response (UPR) is a specific signaling pathway activated, owing to the accumulation of misfolded proteins, in the endoplasmic reticulum (ER) upon different physiological and pathological conditions which endanger ER functions [7].

UPR is driven by three ER-transmembrane transducers, IRE1α (Inositol Requiring 1α), PERK (PKR-like ER Kinase) and ATF6α (Activating Transcription Factor 6α), that under physiologic conditions are kept inactive by the binding with the chaperone BiP/GRP78 (Binding Immunoglobulin Protein/Glucose-Regulated Protein 78) [7–9].

As a consequence of stress, BiP binds the hydrophobic residues of unfolded proteins, to facilitate proper protein folding, to prevent protein aggregate formation and to drive misfolded proteins to the final proteasomal degradation [10]. The ER transducers, then, dissociate from BiP and activate the UPR signal, triggering the activation of their downstream targets eIF2α, ATF4 (Activating Transcription Factor 4 (tax-responsive enhancer element B67)) and XBP1, giving a prompt response to raise protein folding capacity, degrade misfolded proteins and slow down *de novo* protein synthesis [7, 11]. However, when stress is protracted and ER functions are severely impaired, the organelle triggers apoptotic cell death, through a mechanism that has not been completely elucidated and that involves different proteins like the transcription factor CHOP/GADD153 [12], leading to elimination of cells unable to handle the unfolded protein accumulation through the UPR intervention [12–14].

The endocannabinoid system includes the CB receptors, the endocannabinoids and the enzymes involved in their synthesis and degradation, all located in the brain and peripheral tissues, including the liver [15]. It has been widely demonstrated that the endocannabinoid system is involved in a broad range of biological processes, like food intake, energy balance and immune responses, as well as in the pathogenesis of different human diseases including cancer, neurological disorders and cardiovascular disease [16]. In particular, the endocannabinoid system plays an important role in the patho-physiological processes associated with acute and chronic liver diseases, stimulating inflammation, liver damage and fibrogenesis [17, 18]. CB1 and CB2 receptors expression, the responsive elements to endo-cannabinoids, is quite low in normal liver and strongly upregulated in experimental liver injury and cirrhosis due to alcohol, hepatitis and primary biliary cirrhosis [18–20]. Interestingly, some authors demonstrated that cannabinoids are able to activate the ER stress response in different models [21–27].

The aim of our work was to investigate the effects of HBV envelope proteins expression in liver cancer cells, focusing on the activation of ER stress pathway and analyzing the role exerted by the CB receptors in this *scenario*.

## RESULTS

### Effects of pSVL and pSVM plasmids transfection on cell viability

**Figure 1:**
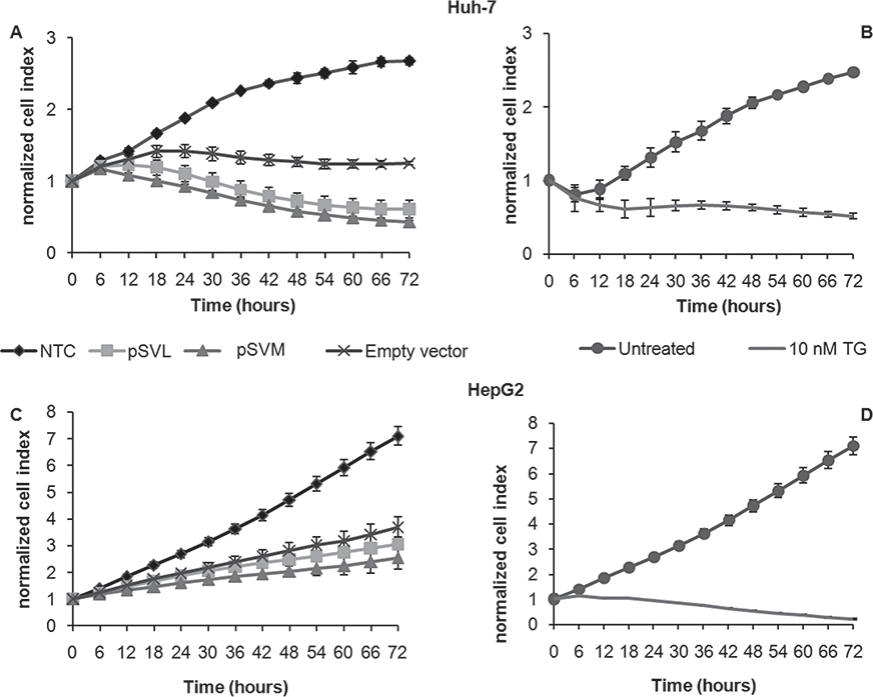
Real-time cell viability analysis after pSVL and pSVM plasmids transfection For the impedance based real time cell viability study, Huh-7 (**A** and **B**) and HepG2 (**C** and **D**) cells were cultured in E-plates, transfected with Empty vector, pSVL or pSVM plasmids or treated with the vehicle only (Untreated), with the transfection reagent only (NTC, Negative control) or TG (10 nM). Cell index was normalized to the time point of treatment. Cell index was determined continuously for 72 hours. Shown are means ± SD of three independent experiments.

We first performed real-time cell viability analysis with the xCELLigence system to assess the effects of pSVL and pSVM plasmids transfection on cell proliferation of Huh-7 and HepG2 cell lines. The results showed that transfection with pSVL and pSVM plasmids caused a strong decrease of normalized cell index in Huh-7 cells, comparable to the effects of 10 nM thapsigargin (TG), a well-known ER stress inducer (Figure 1A and 1B). pSVL and pSVM plasmids transfection induced slight effects on HepG2 cells viability only (Figure 1C and 1D). Flow cytometry analyses after 72 hours transfection with both plasmids showed no variation of cell cycle distribution in both cell lines; a small increase of the percentage of sub-G1 events in Huh-7 cells (Supplementary Figure S1A and S1B) was observed. The following data were acquired after 72 hours of transfection, which is the best transfection efficacy time point as shown by Supplementary Figure S1C and S1D.

### LHBs is localized in the ER compartment

**Figure 2:**
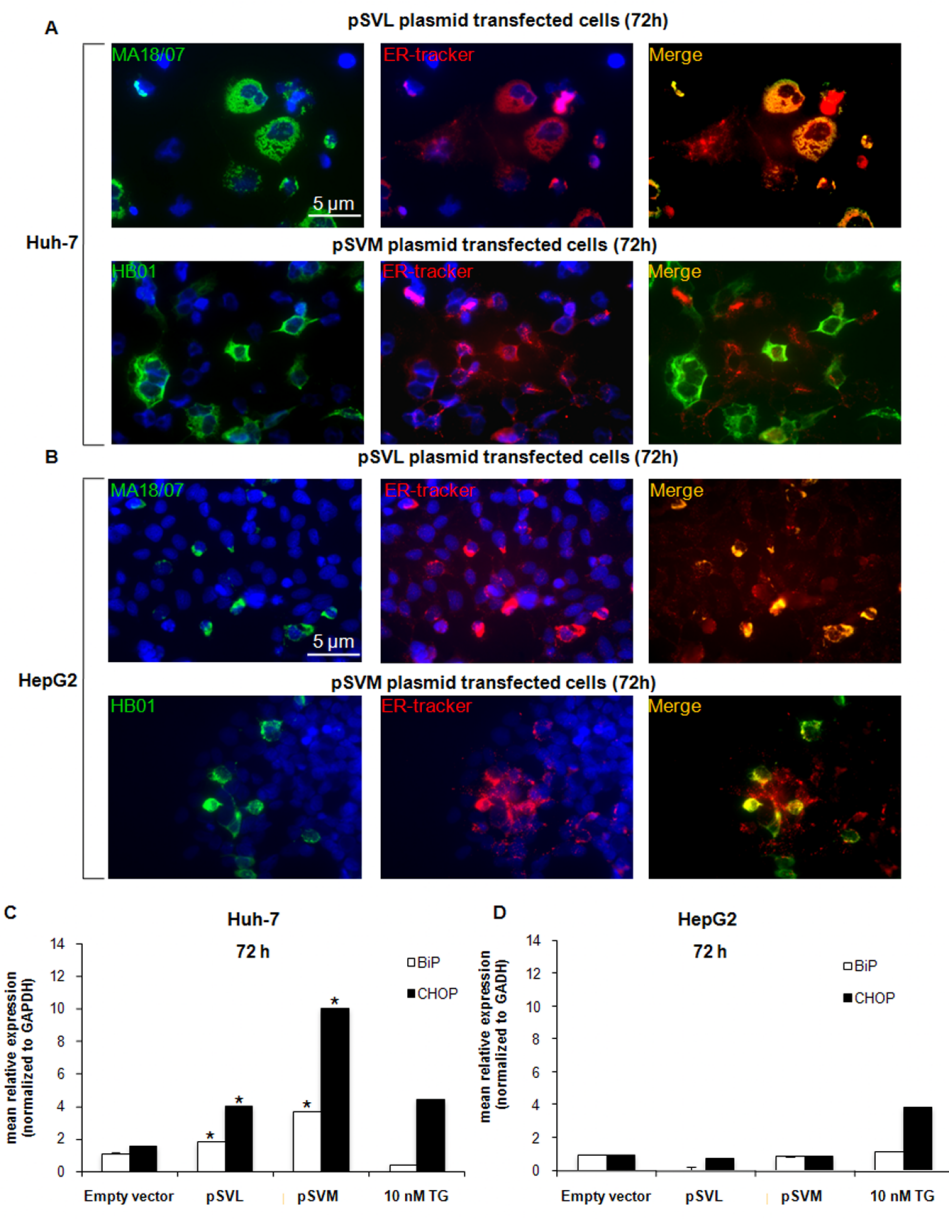
HBV envelope proteins localization and ER stress markers expression Immunofluorescence analysis of LHBs and MHBs proteins co-localization with the ER specific dye ER-Tracker in Huh-7 (**A**) and HepG2 (**B**) cells after transfection with Empty vector, pSVL or pSVM plasmids, or treatment with 10 nM TG for 72 hours. Immunofluorescence analysis has been performed under identical settings. Nuclei were stained with Hoechst 33342. Magnification is 630× and scale bar represents 5 μm. RT-qPCR analysis of BiP and CHOP after 72 hours transfection with Empty vector, pSVL or pSVM plasmids, or treatment with 10 nM TG in Huh-7 (**C**) and HepG2 (**D**) cells. mRNA expression was normalized to GAPDH and results are expressed relative to Empty vector for pSVL or pSVM plasmids transfected cells, and to untreated controls for TG treated cells, and set in both cases at 1.0. Shown are means ± SEM of three independent experiments performed in triplicates. *p < 0.05 vs. Empty vector.

It has been reported that accumulation of HBV envelope proteins could lead to induction of ER stress [5, 39–43] (Bouchard and Navas-Martin, 2011; Chua et al., 2005; Hsieh et al., 2004; Li et al., 2011; Schluter et al., 2001; Su et al., 2008). To analyze LHBs and MHBs localization after pSVL and pSVM plasmids transfection in Huh-7 and HepG2 cells, we performed a double immunofluorescence staining with ER-Tracker, a cell permeable ER highly selective dye, and specific antibodies against LHBs and MHBs proteins. The results clearly showed that the LHBs protein co-localizes with the ER-Tracker in both cell lines, while the MHBs protein is located inside the ER lumen only partly (Figure 2A and 2B). These results were confirmed also after 24 and 48 hours of transfection in both cell lines (data not shown).

### The expression of both HBV envelope proteins up-regulates ER stress-related factors

It has been already reported that viral infection can lead to the activation of stress pathways like ER stress [28, 29]. Here we analyzed, in detail, the expression of the ER stress-related factor IRE1α, the chaperone BiP and the transcription factors ATF4 and CHOP in both cell lines after transfection with pSVL and pSVM plasmids or after 72 hours treatment with 10 nM TG. The expression of BiP and CHOP was significantly increased after 48 hours transfection with both plasmids in Huh-7 cells already (Supplementary Figure S2A) and it was even higher after 72 hours transfection (Figure 2C); while its level was stable in HepG2 cells (Figure 2D and Supplementary Figure S2B). IRE1α and ATF4 levels were almost stable in both cell lines after transfection with both plasmids (Supplementary Figure S2A and S2B). 10 nM TG treatment was assessed as ER stress inducer in both cell lines (Figure 2C and 2D; Supplementary Figure S2A and S2B).

### Activation of ER stress pathway induced by HBV envelope proteins

**Figure 3:**
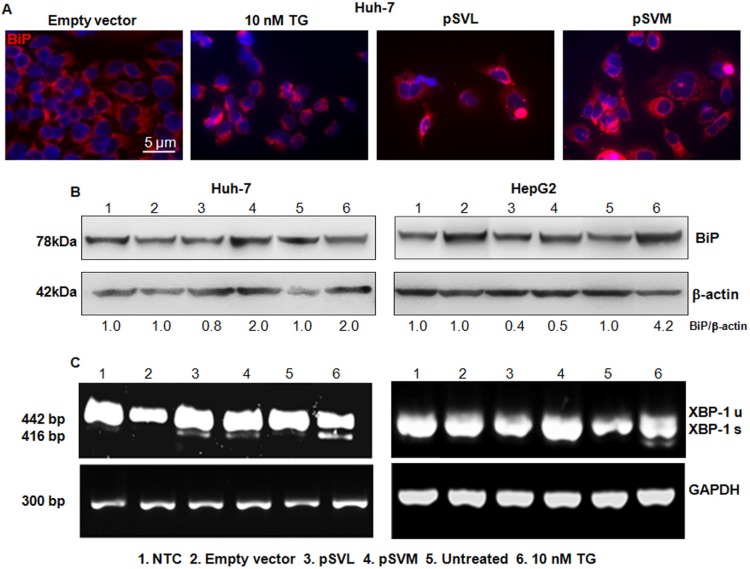
BIP detection and Xbp-1 splicing Immunofluorescence analysis of BiP after 72 hours of transfection with Empty vector, pSVL or pSVM plasmids, or treatment with 10 nM TG in Huh-7 cells (**A**). Immunofluorescence analysis has been performed under identical settings. Nuclei were stained with Hoechst 33342. Magnification is 630 × and scale bar represents 5 μm. Western blot results of BiP in Huh-7 (**B**) left blots) and HepG2 (B, right blots) cells, after 72 hours of transfection with Empty vector, pSVL or pSVM plasmids, or treatment with 10 nM TG. Densitometry results were normalized to β-actin content and are expressed relative to Empty vector for pSVL and pSVM plasmids transfected cells, and to untreated controls for TG treated cells and set in both cases at 1.0. RT-PCR analysis of total RNA isolated from Huh-7 (**C**) lowest left panels) and HepG2 (C, lowest right panels) cells transfected with Empty vector, pSVL or pSVM plasmids, or treated with 10 nM TG for 72 hours. For this experiment, primers amplifying both unspliced (XBP-1u, 442 bp) and spliced (XBP-1s, 416 bp) forms of XBP-1 mRNA were used. Levels of GAPDH mRNA were used as internal control.

We further investigated the status of additional ER stress markers after pSVL and pSVM plasmids transfection, to better elucidate the exact mechanisms induced by HBV envelope proteins in HCC cell lines. BiP protein level was evaluated in Huh-7 cells by Immunofluorescence and Western blot analysis after plasmid transfection or treatment with TG (Figure 3A and 3B; Supplementary Figure S3A). The immunofluorescence results showed the increase of BiP protein in Huh-7 transfected cells; the effect was comparable to 10 nM TG (Figure 3A; Supplementary Figure S3A). CHOP protein level was analyzed in Huh-7 cells by Immunofluorescence also, showing stable level after plasmid transfection (Supplementary Figure S3B). Furthermore, western blot data revealed an increased level of BiP after transfection with pSVM plasmid (2.0 fold increase, as quantified by densitometric analysis) and a stable level after transfection with pSVL plasmid in Huh-7 cells (Figure 3B).

In HepG2 cells, BiP was evaluated by Western blot analysis also, showing a decrease after transfection with both plasmids (0.4 and 0.5 fold increase, Figure 3B). TG caused the increase of BiP protein level in both cell lines (Figure 3B).

### HBV envelope proteins induced XBP-1 splicing and activated the ER stress sensor PERK

Under physiological condition, the chaperone BiP binds IRE1α, PERK and ATF6α and keeps them inactive. Accumulation of unfolded proteins attracts BiP that releases the three ER transducers. Once free, IRE1α auto-phosphorylates, and catalyzes the splicing of XBP-1 to generate an active transcription factor [9, 30]. We, then, analyzed IRE1α/XBP-1 arm involvement through XBP-1 splicing and we found that XBP-1 was spliced in Huh-7 cells but not in HepG2 cells after plasmids transfection (Figure 3C, lane 3 and 4). TG, always used as a positive control of ER stress, induced the splicing of XBP-1 in both cell lines (Figure 3C, lane 6). After dissociation from BiP, PERK can dimerize, auto-phosphorylate and reach its active kinase status. PERK then phosphorylates and inhibits eIF2α, leading to a selective synthesis of ATF4 and CHOP [31]. In order to further confirm ER stress pathway induction in Huh-7 cells, we analyzed by Immunofluorescence the status of PERK and phospho-eIF2α after transfection with pSVL and pSVM plasmids or treatment with TG. The results showed strong increase of both PERK and phosphoSer51-eIF2α after expression of both ectopic HBV envelope proteins. Treatment with 10 nM TG induced also a high increase of PERK and phosphoSer51-eIF2α (Supplementary Figure S4A and S4B).

### ER stress activation induced by HBV envelope proteins depends on CB1 expression

**Figure 4:**
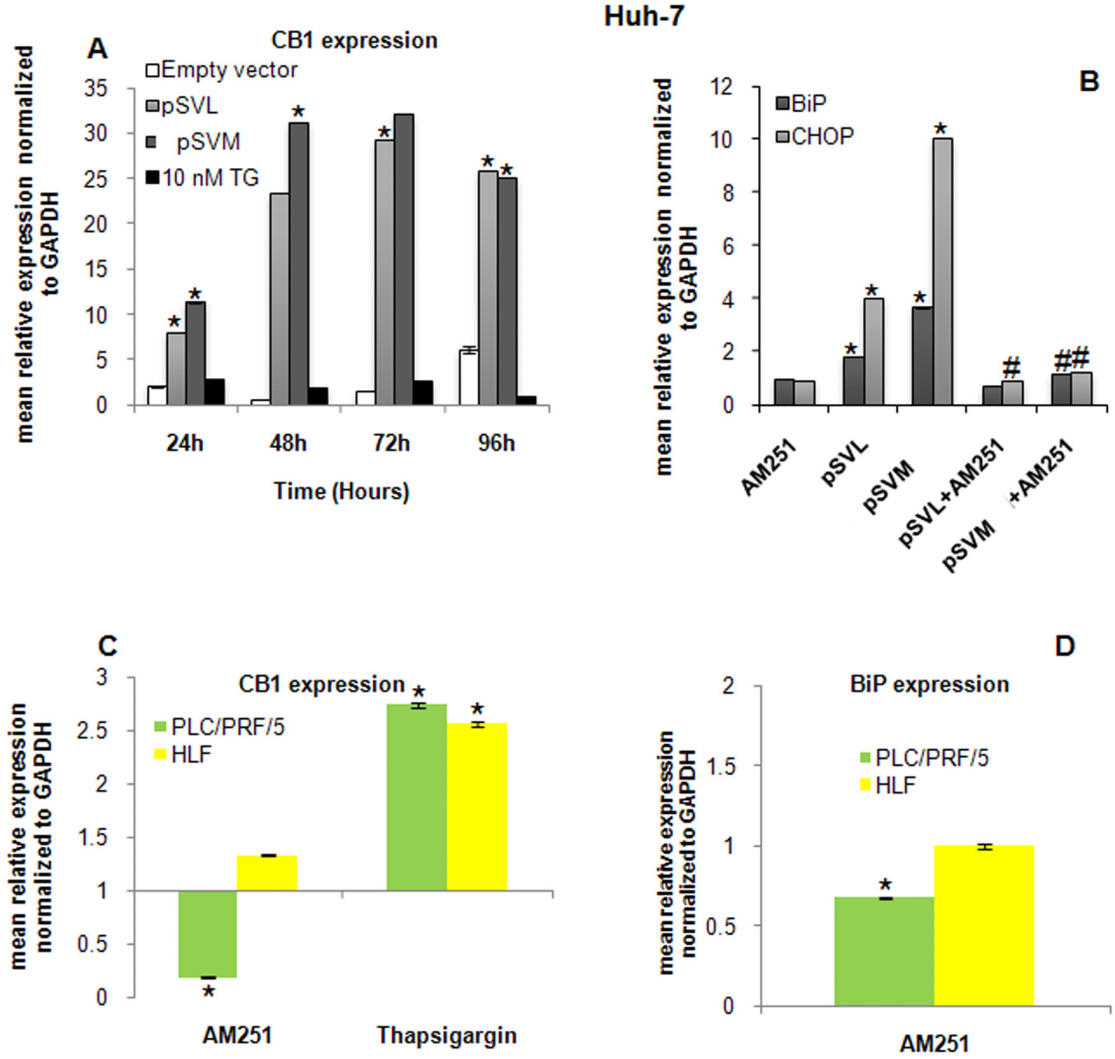
Role of CB1 in ER stress induction (**A**) RT-qPCR analysis of CB1 expression after 24, 48 72 and 96 hours transfection with Empty vector, pSVL or pSVM plasmids, or treatment with 10 nM TG in Huh-7 cells. mRNA expression was normalized to GAPDH and results are expressed relative to Empty vector for pSVL and pSVM plasmids transfected cells, and to untreated controls for TG treated cells and both set at 1.0. Shown are means ± SEM of three independent experiments performed in triplicates. *p < 0.05 vs. Empty vector. BiP and CHOP expression has been analyzed in Huh-7 cells (**B**) after 72 hours transfection with Empty vector, pSVL or pSVM plasmids and/or treatment with the cannabinoid receptor antagonist AM251. mRNA expression was normalized to GAPDH and results are expressed relative to Empty vector for pSVL and pSVM plasmids transfected cells, and to untreated controls for TG treated cells and both set at 1.0. Shown are means ± SEM of three independent experiments performed in triplicates. *p < 0.05 vs. control, ^#^*p* < 0.05 vs. pSVL or pSVM transfected cells. (**C**) RT-qPCR analysis of CB1 expression after 72 hours treatment with 10 nM AM251 and 10 nM TG in PLC/PRF/5 and HLF cells. (**D**) BiP transcript was detected in PLC/PRF/5 and HLF cells after 72 hours treatment with 10 nM AM251 and 10 nM TG. mRNA expression was normalized to GAPDH and results are expressed relative to untreated set at 1.0. Shown are means ± SEM of three independent experiments performed in triplicates. *p < 0.05 vs. control.

It has been demonstrated that the endocannabinoid system is able to modulate the ER stress response and to activate some markers of ER stress. We further analyzed the involvement of CB1 receptor in ER stress induced by HBV envelope proteins. First, we evaluated by PCR the expression level of CB1 in Huh-7 cell line after plasmids transfection or after TG treatment (Figure 4A). CB1 expression strongly and significantly increased (*p < 0.05), already after 24 hours transfection with LHBs and MHBs plasmids; its level was stably high for 96 hours transfection (Figure 4A). TG treatment did not modulate CB1 expression. CB1 level was evaluated in HepG2 cells also, but its expression was not detectable [32] (data not shown).

It has been widely demonstrated that CB receptors can activate the ER stress response in different models [15, 24, 27]. However, the underlying molecular and cellular mechanisms that promote the activation of this signaling pathway remain elusive. In our model, HBV envelope proteins were able to trigger the ER stress response in Huh-7 cells, in particular increasing BiP and CHOP levels (Figure 2C), and to highly induce CB1 expression (Figure 4A). In order to elucidate a possible involvement of CB1 in ER stress induction after plasmids transfection, we used a potent CB1 receptor antagonist, AM251 [33]. Interestingly, CB1 inhibition strongly and significantly counteracts BiP and CHOP expression, neutralizing the effects of HBV envelope proteins on these ER stress markers (Figure 4B).

Furthermore, two additional HCC cell lines PLC/PRF/5 and HLF were included in the study in order to confirm the role exerted by CB1 receptor during HBV infections. It is well known that HBV is integrated at the genome of PLC/PRF/5 cells and they secrete HBsAg [34], whereas HBV is not detectable in HLF cells [35]. Expression of CB1 receptor transcript was confirmed in both cell lines by RT-qPCR (Figure 4C). Interestingly, the addition of the CB1 receptor antagonist AM251 caused a significant decrease of CB1 receptor expression in the HBV infected cells PLC/PRF/5, whereas its expression was unvaried in HLF cells. Inhibition of CB1 receptor by 72 hours treatment with AM251 caused a significant down-regulation of BiP transcript in PLC/PRF/5 cells, as shown in Huh-7 cells. HLF cells, negative for HBV, did not show any variation in the level of BiP transcript (Figure 4D). CHOP transcript was not detectable in both cell lines.

Interestingly, treatment with 10 nM TG for 72 hours, used here as positive control as for Huh-7 cells, induced a significant over-expression of CB1 receptor transcript in both cell lines (Figure 4C). These results confirm the role exerted by HBV and its interaction with CB1 receptor in ER stress-related *scenario*.

## DISCUSSION

HCC is one of the most frequent solid tumors occurring worldwide and chronic infection with HBV and HCV, especially in the setting of advanced cirrhosis or fibrosis, have been identified as the leading causes of HCC [36, 37]. HBV is a small DNA virus, a member of the hepadnaviridae family, with a genome that contains four overlapping open reading frames, encoding for the core protein, for the viral polymerase, for the HBx protein, known to be involved in HBV-associated carcinogenesis, and for the envelope proteins. Specifically, HBV genome owns three co-carboxyterminal HBV envelope proteins, named LHBs, MHBs and SHBs proteins that differ for their distinct domains and glycosylation status [38]. HBV infection can lead to HCC development through direct mechanisms, including viral DNA integration into host genome, transcriptional activation of various cellular genes due to HBx protein or truncated MHBs [39], and overexpression of envelope proteins, and/or through indirect processes, like inflammation, regeneration and fibrosis associated with cirrhosis [36]. In particular, excessive production of HBV envelope proteins LHBs and SHBs or mutated/truncated forms of LHBs, MHBs and SHBs, can activate cellular signal stress response pathways, like ER stress, that alter hepatocyte physiology and may stimulate neoplastic processes [5, 39–44].

The accumulation of unfolded or misfolded proteins inside the ER, due to many different perturbations of this organelle homeostasis, leads to a cellular condition known as ER stress that triggers a series of adaptive mechanisms, named unfolded protein response (UPR), designed to restore standard protein folding [45, 46] and orchestrated by three signaling proteins, IRE1α, PERK and ATF6. If ER stress is chronically protracted, cells undergo cell death and one of the main player of ER stress-mediated apoptosis is the transcription factor CHOP [46]. UPR is activated in several acute and chronic liver diseases and the activation of ER-stress downstream molecules such as ATF-6, BiP and XBP-1 is involved in hepatocarcinogenesis [7, 47].

Despite the huge amount of data demonstrating a connection between HBV infection and HCC development and the great breakthrough in this field, the precise underlying processes and molecular pathways linking HBV and HCC are not completely highlighted yet. The major obstacle is the lack of *in vitro* and *in vivo* experimental models that reliably mimic human HBV infection [5, 37]. In our study, we used Huh-7 and HepG2 cell lines that were transiently transfected with plasmids encoding for HBV envelope proteins; the first one was expressing LHBs and minor amounts of MHBs and SHBs proteins and the second one expressing MHBs alone. The proposed models were used to analyze ER stress path.

Here, we show that pSVM and pSVL plasmids are able to trigger an ER stress response in Huh-7 cells, comparable to the effects of the well known ER stress inducer thapsigargin, upregulating the chaperone BiP and the transcription factor CHOP, two gold standard ER stress markers [48], probably as a consequence of HBV envelope proteins accumulation inside the ER compartment as shown by the detection of ER-Tracker, a specific ER dye. On the contrary, none of the ER stress factors analyzed are activated in HepG2 cells, but they are rather reduced after HBV envelope proteins exogenous expression. One of the ER stress mediator, IRE1α is involved in the processing of XBP-1 mRNA and in the recruitment of TRAF2/ASK1 (TNF receptor-associated factor 2/Apoptosis signal-regulating kinase 1) to mediate the activation of JNK (c-Jun N-terminal kinase) and of nuclear factor kappa B (NF-kB) [9, 49]. XBP-1 spliced form can induce the expression of several genes involved in different aspects of UPR [9, 12]. XBP-1 splicing was clearly detectable in Huh-7 cells, but not in HepG2 after expression of HBV envelope proteins, strengthening that HBV envelope proteins are able to trigger an ER stress response only in Huh-7 cells.

PERK, one of the three ER stress sensors, activated after BiP dissociation, is the major protein responsible for attenuation of mRNA translation under ER stress. Once activated, it phosphorylates eIF2α, inhibiting the initiation phase of polypeptide chain synthesis and allowing the preferential translation of UPR-dependent genes, such as ATF4 [46]. HBV envelope proteins expression leads to the increase of PERK and phospho-eIF2α level in Huh-7 cells, confirming the activation of ER stress pathway.

Interestingly, it has been shown a link between ER stress induction and the endocannabinoid system [15]. The cannabinoids are a group of different lipidic compounds, acting via the G-protein-coupled receptors CB1 and CB2 and other putative targets, playing a role in a wide range of cellular processes, like food intake, energy balance, nociception, intestinal motility and immune responses [17]. Moreover, the cannabinoids play a role in inflammatory processes and cancer progression. In particular, it has been shown that cannabinoids are strongly involved in liver diseases and related pathophysiological conditions, as altered hepatic haemodynamics, cirrhotic cardiomyopathy, metabolic syndrome and ischaemia/reperfusion disease [17, 18]. The endocannabinoids synthesis and CB1 and CB2 hepatic expression have been demonstrated to be increased in several conditions characterized by chronic liver damage, such as alcoholic and non-alcoholic fatty liver, viral hepatitis and fibrosis [18–20, 50, 51]. Furthermore, it has been reported that activation of CB1 or CB2 receptors can modulate the activation of ER stress pathway in human tumors including glioma, leukemia, pancreatic and colorectal cancer [15]. Specifically, CB1 or CB2 receptors are able to stimulate *de novo* synthesis of ceramide, a pro-apoptotic lipid that causes upregulation of the stress protein p8 (also named candidate of metastasis 1, Com-1) and its downstream target, the pseudokinase TRB3 (tribbles homologue-3), and several other downstream ER stress-related genes, like ATF4 and CHOP [23–25]. In our study, we also analyzed the possible involvement of CB1 receptor in ER stress induced by HBV envelope proteins. Here, we show that CB1 is highly upregulated after HBV envelope proteins expression in Huh-7 cells, while its level is not detectable in HepG2 cells [32]. Inhibition of CB1 using a specific CB1 receptor antagonist, AM251, counteracts the effects of HBV envelope proteins expression on ER stress induction, restoring BiP and CHOP basal levels, strongly supporting the hypothesis that CB1 can modulate the ER stress response in our model. In HepG2 cells, where CB1 is weakly expressed, the HBV envelope proteins are not able to induce the ER stress pathway. Additionally, the block of CB1 receptor caused a significant down-regulation of BiP transcript level in HBV infected PLC/PRF/5 cells, while no variation of BiP was observed in HBV negative HLF cells. It has been reported that CB receptors can inhibit the serine/threonine protein kinase Akt, thus preventing ER stress blocking and, instead, leading to its activation [22, 24, 26].

In summary, based on our findings, we show that HBV envelope proteins trigger the ER stress response, activating two arms of UPR mechanism, specifically PERK/eIF2α and IRE1α/XBP-1. We could speculate that CB1 expression, in cells infected by HBV, could counteract the inhibitory action of Akt on ER stress pathway and trigger proliferation arrest through ER stress; while the absence of CB1 in HepG2 could not block the survival mechanism of Akt.

In conclusion, we could confirm that ER stress plays an important role in cell transformation, especially in HCC and in chronic viral hepatitis B infection in the proposed model. Furthermore the mechanistic role of cannabinoid system, a crucial checkpoint in liver diseases, would need to be clarified in correlation with ER stress after HBV infection.

## MATERIALS AND METHODS

### Cell culture and reagents

Human HCC cells Huh-7, PLC/PRF/5 and HLF were cultured in Dulbecco's modified Eagle's medium (DMEM) (Biochrom, Berlin, Germany) supplemented with 10% FBS (Biochrom) and HepG2 were cultured under standard conditions as previously described [8]. Cells were transfected with plasmid DNA using FuGENE^®^ HD Transfection Reagent (Promega GmbH, Mannheim, Germany) according to the manufacturer's instructions and harvested for further experiments after 72 hours of transfection. Where indicated, cells were treated, before harvesting, with 10 nM thapsigargin, purchased from Sigma-Aldrich (Munich, Germany) and with 10 nM AM251, purchased from R & D Systems Gmbh (Wiesbaden-Nordenstadt, Germany) and dissolved in dimethyl sulfoxide (DMSO; Sigma-Aldrich).

### Plasmids

The plasmid pSVL (kindly provided by Dr. Volker Bruss from Helmholtz Center Munich, Munich, Germany), encodes for the whole HBsAg, overexpresses LHBs and produces minor amounts of MHBs and SHBs proteins; the plasmid pSVM (kindly provided by Dr. Bruss) encodes for MHBs; the empty vector pGL2- Empty control -SV40-Luc (plasmid 26280, Addgene, USA) has been used as a negative control.

### Real-time cell viability analysis

The xCELLigence RTCA SP system (Roche Applied Science, Mannheim, Germany) was used for real-time analysis of Huh-7 and HepG2 cells viability following transfection with the plasmids pSVL and pSVM and incubation with 10 nM thapsigargin as previously described [52]. Cell index, indicating attachment and adherence of cells to the plate's electrode, was measured continuously for the following 72 hours. Data analysis was performed using the RTCA Software v1.2.1.

### Quantitative and semi-quantitative RT-PCR

For semi-quantitative PCR and quantitative real time PCR, total cellular RNA was extracted using the RNeasy Mini Kit (QIAGEN, Hilden, Germany) according to the manufacturer's instructions and reverse transcription (RT) was performed with Quantitect Reverse Transcription Kit (QIAGEN). For semi-quantitative PCR, the Ready Mix Taq PCR kit (Sigma-Aldrich) was used. The oligonucleotides 5′-CCTTGTAGTTGAGAACCAGG-3′ and 5′-GGGGCTTGGTATATATGTGG-3′ (Eurofins MWG Operon, Ebersberg, Germany) were used for amplification of the XBP-1 transcript fragments. PCR products were resolved on 2% agarose gels, stained with Sybersafe DNA gel stain (Life Technologies, Darmstadt, Germany) and visualised under ultraviolet illumination using Fusion image capture (PEQLAB Biotechnologie GmbH, Erlangen, Germany). Glyceraldehyde-3-phosphate dehydrogenase (GAPDH; 5′-GTCGTGGATCTGACGTGCC-3′ and 5′-GATGCCTGCTTCACCACCTT-3′) was amplified as internal control. For quantitative real time PCR, QuantiTect Primers for IRE1α, BiP, ATF4, CHOP, CB1 and GAPDH were purchased from QIAGEN and run with the QuantiFast SYBR Green PCR Kit (QIAGEN) on a CFX96 Real Time PCR Detection System (BioRad, Munich, Germany). Results were analysed with the CFX Manager v2.0 and Rest 2008 software and normalised to GAPDH mRNA content for each sample.

### Immunofluorescence

Huh-7 and HepG2 cells were seeded in chamber slides (Lab TekTM distributed by Fisher Scientific GmbH, Schwerte, Germany), transfected with pSVL and pSVM plasmids or treated with 10 nM thapsigargin for 72 hours. Immunofluorescence was performed as previously described [52]. Primary antibody against BiP was purchased from Abcam (Cambridge, UK) and secondary AlexaFluor 568-conjugated antibody from Life Technologies. The HBV envelope proteins were detected with the following antibodies: the mouse monoclonal antibody MA 18/07 (anti-preS1), used to detect the LHBs protein [53], and HB01 (anti-SHBs) used to detect MHBs protein, were a kind gift of Dr. Glebe and Dr. Aurelija Zvirbliene, (Institute of Biotechnology, University of Vilnius, Lithuania), respectively. To examine the ER compartment, Huh-7 and HepG2 cells were stained with 1 μM ER-tracker Red (Glibenclamide BODIPY TR, Life Technologies) according to the manufacturer's instructions and detected by Immunofluorescence. The fluorescence was visualized with a Nikon microscope at 630 × magnification and acquired with a Hamamatsu ORCA-ER camera (model C4742–80) under the same setting. Obtained data were analysed with ImageJ software v 1.43u.

### Protein extraction and western blot analysis

Whole cell lysates were obtained from Huh-7 and HepG2 cell lines with or without transfection with pSVL and pSVM and 10 nM thapsigargin after 72 hours, and further processed by SDS-Page followed by western blotting, as previously described [52]. Immunodetection was performed with primary antibodies against BiP, (Abcam) and β-actin (Sigma-Aldrich). Secondary HRP-conjugated antibodies (Sigma-Aldrich) were detected by incubating the immunoblots with SuperSignal West Pico Chemiluminescent Substrate (Pierce, Thermo Fisher Scientific). The luminescent reactivity was measured by using Fusion image capture and further quantified with Bio1D analysis system (PEQLAB Biotechnologie GmbH). Anti β-actin was used as equal loading control and protein quality.

### Statistical analysis

Statistical analysis was performed using SPSS 15.0.1 for Windows (SPSS Inc., Chicago, IL, USA). *P* < 0.05 was regarded as significant.

## SUPPLEMENTARY MATERIAL FIGURES



## Abbreviations

HCC: Hepatocellular carcinoma
HBV: Hepatitis B virus
ER: Endoplasmic Reticulum
CB: Cannabinoid.
