# Supplementary material for: Exogenous hepatitis B virus envelope proteins induce endoplasmic reticulum stress: involvement of cannabinoid axis in liver cancer cells
Source: Oncotarget. 2016 Mar 7;7(15):20312–23. doi: 10.18632/oncotarget.7950 (PMC4991457; doi:10.18632/oncotarget.7950)
Supplement: Supplementary file 1 [file oncotarget-07-20312-s001.pdf]

Exogenous hepatitis B virus envelope proteins induce endoplasmic reticulum stress: involvement of cannabinoid axis in liver cancer cells

Supplementary Materials

SUPPLEMENTARY MATERIALS AND METHODS

Immunofluorescence

Huh-7 and HepG2 cells were seeded in chamber slides (Lab TekTM distributed by Fisher Scientific GmbH, Schwerte, Germany) and transfected with pSVL and pSVM plasmids for 72 hours. The HBV envelope proteins were detected with the following antibodies: the mouse monoclonal antibody MA 18/07 (anti-preS1), used to detect the LHBs protein (Ren and Nassal, 2001), and HB01 (anti-SHBs), used to detect MHBs protein, were a kind gift of Dr. Glebe from the University of Giessen, Germany and Dr. Aurelija Zvirbliene, (Institute of Biotechnology, University of Vilnius, Lithuania), respectively. Primary antibodies against BiP, CHOP, PERK and Ser51-eIF2α were purchased from Abcam (Cambridge, UK) and secondary AlexaFluor488- and 568-conjugated antibodies from Life Technologies (Darmstadt, Germany). Immunofluorescence was performed as previously described (Di Fazio et al., 2012).

Flow cytometry

Flow cytometry was employed for transfection efficiency establishment in Huh-7 and HepG2 cells transfected for 72 hours with pSVL and pSVM plasmids. MA 18/07 and HB01 antibodies were used to detect HBV envelope proteins. Secondary AlexaFluor488-conjugated antibody was purchased from Life Technologies and used as negative control to perform the fluorescence setting also. Flow cytometry was employed for cell cycle analysis also in plasmid transfected or thapsigargin treated cell lines for 72 hours after staining with propidium iodide as described previously (Di Fazio et al., 2010). Analysis of labeled cells and nuclei was performed on an Attune acoustic focusing cytometer (Applied Biosystems, Carlsbad, USA) and results were analyzed with the Attune® Cytometric Software 1.2.5.3891. Twenty thousand events were collected for each sample for transfection efficiency analysis. Ten thousand events were collected for each sample for cell death analysis. All experiments were performed in triplicates.

Quantitative RT-PCR

For real time PCR, total cellular RNA was extracted using the RNeasy Mini Kit (QIAGEN, Hilden, Germany) according to the manufacturer's instructions and reverse transcription (RT) was performed with Quantitect Reverse Transcription Kit (QIAGEN). QuantiTect Primers for IRE1α, BiP, ATF4, CHOP, CB1 and GAPDH were purchased from QIAGEN and run with the QuantiFast SYBR Green PCR Kit (QIAGEN) on a CFX96 Real Time PCR Detection System (BioRad, Munich, Germany). Results were analyzed with the CFX Manager v2.0 and Rest 2008 software and normalized to GAPDH mRNA content for each sample.

SUPPLEMENTARY RESULTS

Cell cycle analysis distribution

To assess the effects of pSVL and pSVM plasmids transfection on cell cell cycle distribution, we performed flow cytometry analyses after 72 hours transfection in Huh-7 and HepG2 cell lines. Both plasmids showed any significant variation of cell cycle phases in both cell lines, and only a small increase of the percentage of sub-G1 events in Huh-7 cells (Supplementary Figure S1A and S1B).

Transfection efficiency evaluation

In order to establish the highest transfection efficiency, Huh-7 and HepG2 cells have been transfected with pSVL and pSVM plasmids for 96 hours and Immunofluorescence and Flow cytometry analysis have been performed. Here we show the results after 72 hours transfection, time in which the highest transfection efficiency has been achieved (Supplementary Figure S1C and S1D). In Huh-7 cells, around 40% of cells were positive to MA18/07 antibody, specific for LHBs protein, and to HB01 antibody, specific for MHBs protein (Supplementary Figure S1C); in HepG2 cells, around 30% of cells were positive to MA18/07 antibody, and around 15% were positive to HB01 antibody (Supplementary Figure S1D).

The expression of both HBV envelope proteins upregulates ER stress-related factors

We analyzed the expression levels of the ER stress-related factor IRE1α, of the chaperone BiP and of the transcription factors ATF4 and CHOP in both cell lines after transfection with pSVL and pSVM plasmids or after treatment with 10 nM TG for 72 hours. The expression of BiP and CHOP was significantly induced already after 48 hours transfection with both plamids in Huh-7 cell line (Supplementary Figure S2A) and it increased significantly after 72 hours transfection (Figure 2C); while its level was stable in HepG2 cells (Figure 2D and Supplementary Figure S2B). IRE1α and ATF4 levels were almost stable in both cell lines after transfection with both plasmids (Supplementary Figure S2A and S2B). ER stress markers were confirmed to be strongly upregulated after 10 nM TG treatment, which was used as positive control of ER stress induction (Figure 2C and 2D; Supplementary Figure S2A and S2B).

Analysis of CHOP protein level

The PCR results showed that both pSVL and pSVM transfection were able to strongly and significantly upregulate CHOP in Huh7 cells (Figure 2A). CHOP protein level was analyzed in Huh7 cells by Immunofluorescence, showing an increase and nuclei localization after transfection with both plasmids comparable to the effects of 10 nM TG (Supplementary Figure S2).

HBV envelope proteins activated PERK/eIF2α arm of ER stress

In order to further confirm ER stress pathway induction in Huh-7 cells, we analyzed by Immunofluorescence the status of PERK and phospho-eIF2α after transfection with pSVL and pSVM plasmids or treatment with TG. The results showed a strong increase of both PERK and Ser51-eIF2α after expression of both HBV envelope proteins. Treatment with 10 nM TG induced also a high increase of PERK and Ser51-eIF2α (Supplementary Figure S4A and S4B).

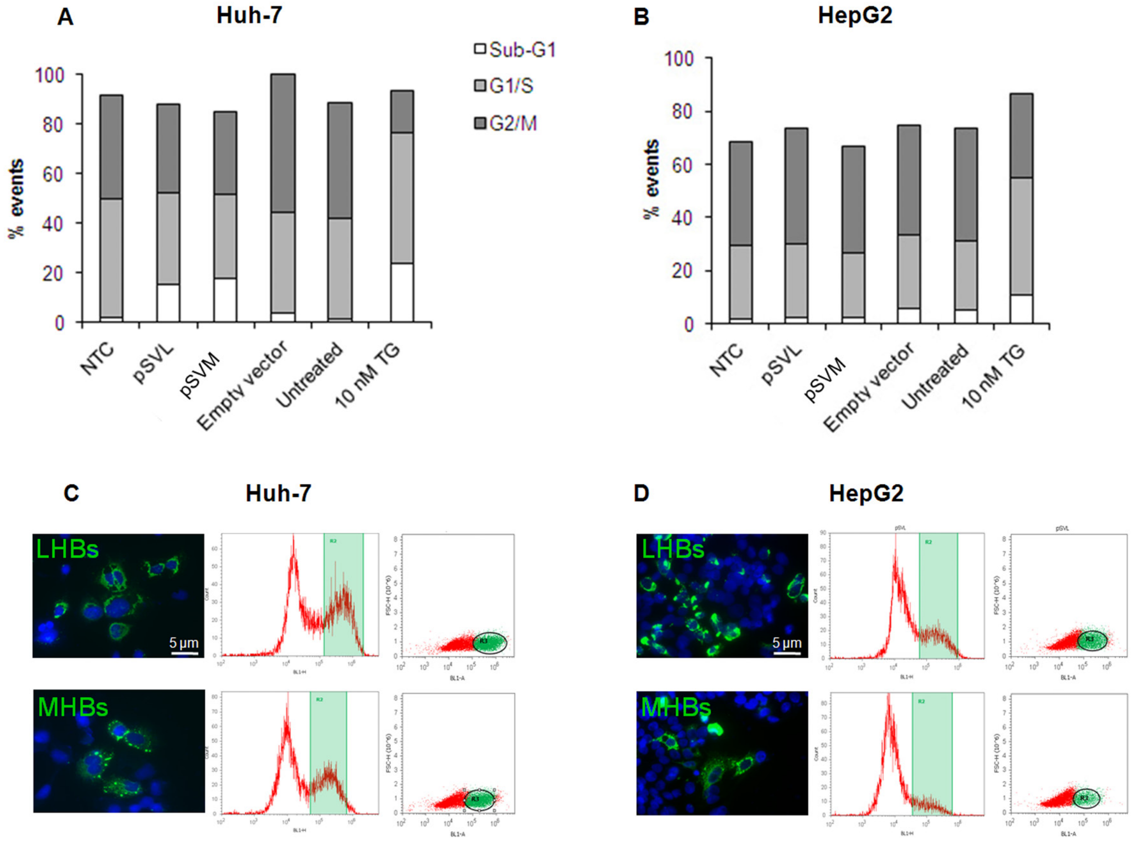

Supplementary Figure S1: Cell cycle distribution analysis. Flow cytometry was employed for cell cycle analysis in transfected or TG treated cell lines for 72 hours after staining with propidium iodide (**A** and **B**). Shown are the percentage of Sub-G1, G1/S and G2/M events. The experiment has been performed in triplicates. **Transfection efficiency evaluation through Immunofluorescence and Flow citometric analyses**. Immunofluorescence analysis of LHBs and MHBs HBV envelope proteins after 72 hours of transfection with pSVL or pSVM plasmids in Huh-7 (**C**) left panels) and HepG2 cells (**D**) left panels). Immunofluorescence analysis has been performed under identical settings. Nuclei were stained with Hoechst 33342. Magnification is 630 × and scale bar represents 5 µm. Flow cytometry has been used to quantify the number of Huh-7 and HepG2 cells transfected for 72 hours with pSVL or pSVM plasmids. The experiment has been performed in triplicates.

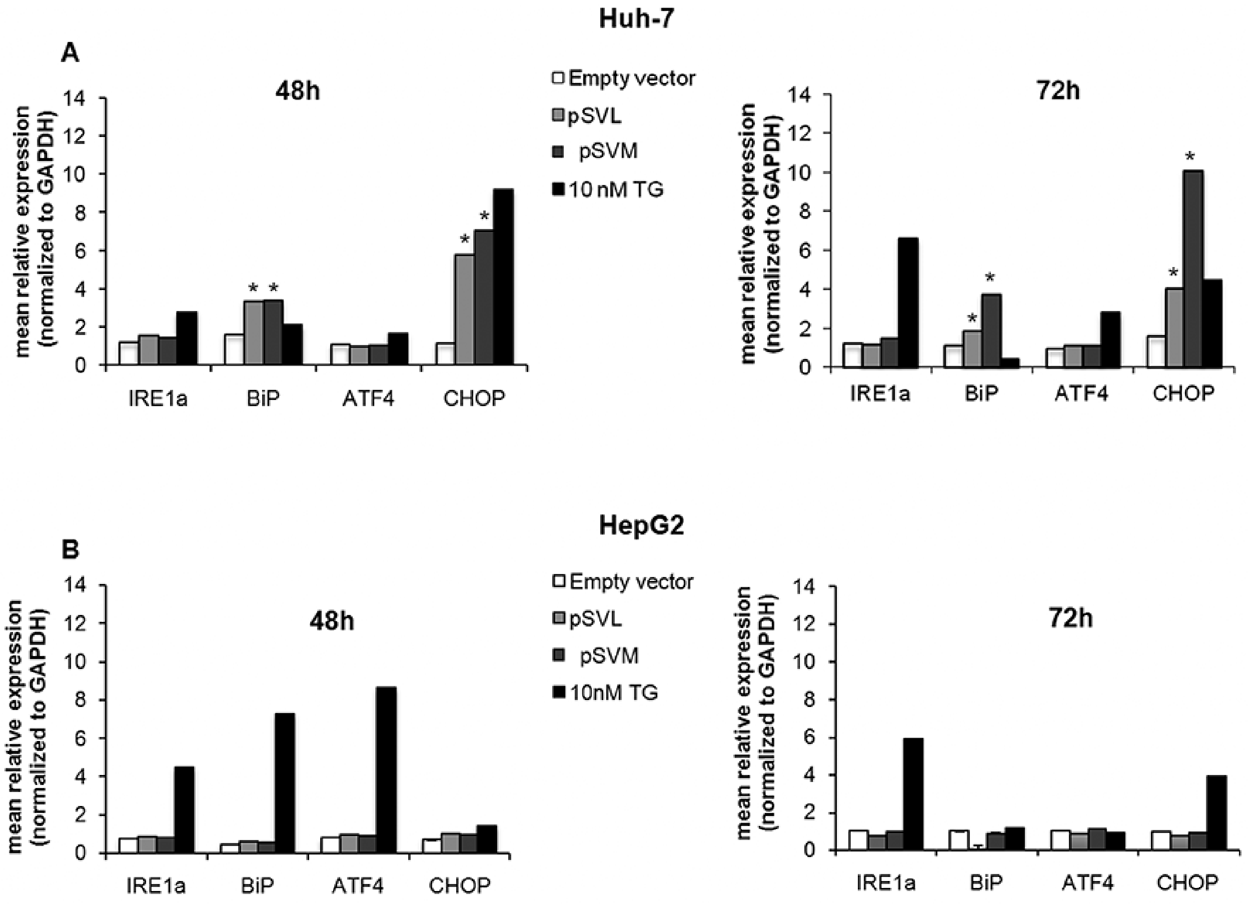

Supplementary Figure S2: mRNA expression of ER stress-related factors. RT-qPCR analysis of IRE1α, BiP, ATF4 and CHOP after 48 and 72 hours transfection with Empty vector, pSVL or pSVM plasmids, or treatment with 10 nM TG in Huh-7 (**A**) and HepG2 (**B**) cells. mRNA expression was normalized to GAPDH and results are expressed relative to Empty vector for pSVL or pSVM plasmids transfected cells, and to untreated controls for TG treated cells, and set in both cases at 1.0. Shown are means ± SEM of three independent experiments performed in triplicates. *$p < 0.05$ vs Empty vector.

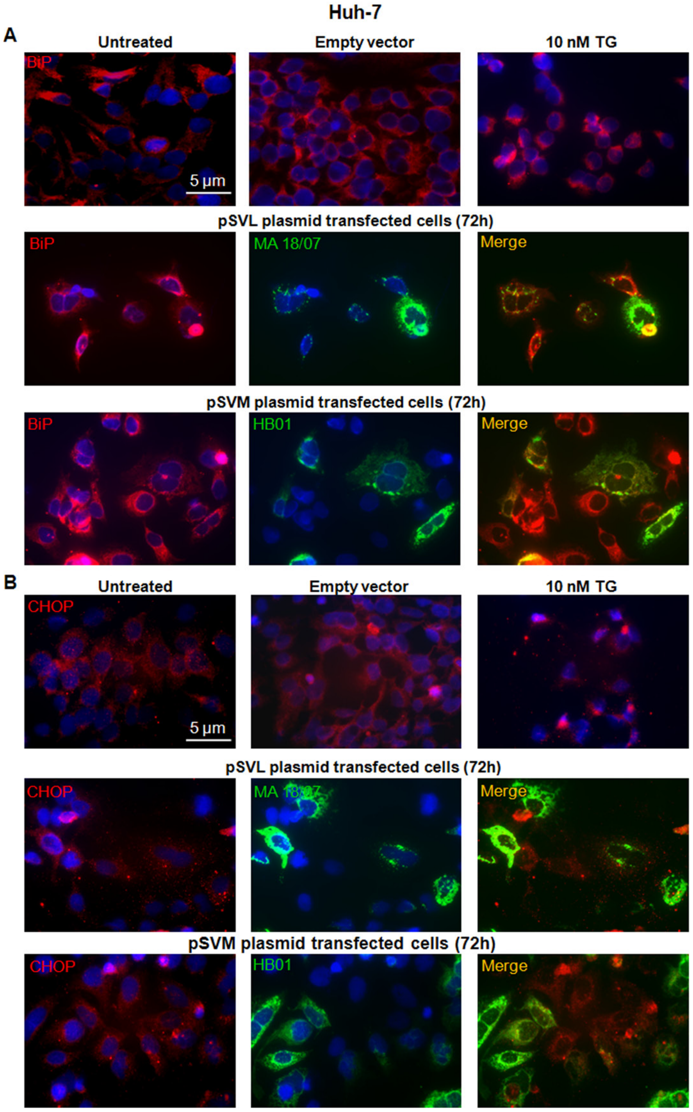

Supplementary Figure S3: BiP (A) and CHOP (B) protein level analysis. Immunofluorescence analysis of BiP and CHOP after 72 hours transfection with Empty vector, pSVL or pSVM plasmids and/or treatment with 10 nM TG in Huh-7 cells. Immunofluorescence analysis has been performed under identical settings. Nuclei were stained with Hoechst 33342. Magnification is 630 × and scale bar represents 5 µm.

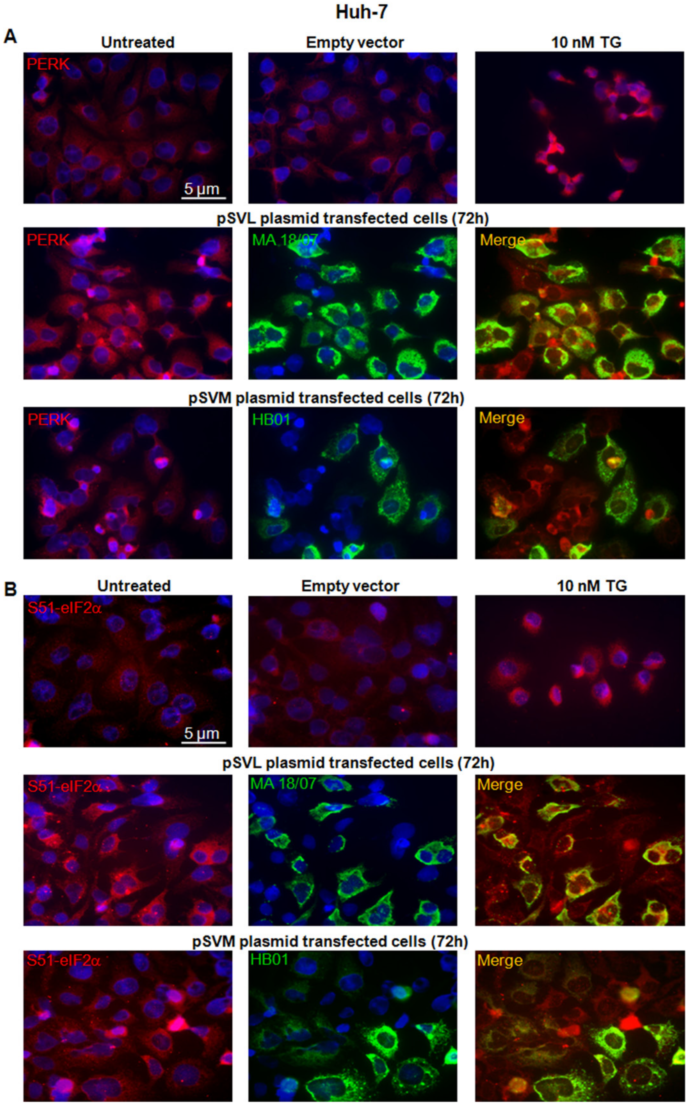

Supplementary Figure S4: PERK (A) and Ser51-eIF2α (B) protein level analysis. Immunofluorescence analysis of PERK and Ser51-eIF2α after 72 hours transfection with Empty vector, pSVL or pSVM plasmids and/or treatment with 10 nM TG in Huh-7 cells. Immunofluorescence analysis has been performed under identical settings. Nuclei were stained with Hoechst 33342. Magnification is 630 × and scale bar represents 5 µm.
